# Effects of Selective Serotonin Reuptake Inhibitor Treatment on Ovarian Reserves in Patients with Depression

**DOI:** 10.3390/medicina59030517

**Published:** 2023-03-07

**Authors:** Soner Gök, Berfin Can Gök, Erkan Alataş, Hande Senol, Osman Zülkif Topak

**Affiliations:** 1 Department of Obstetrics and Gynecology, School of Medicine, Pamukkale University, Denizli 20160, Turkey; 2 Department of Obstetrics and Gynecology, Denizli State Hospital, Denizli 20010, Turkey; 3 Department of Biostatistics, School of Medicine, Pamukkale University, Denizli 20160, Turkey; 4 Department of Psychiatry, School of Medicine, Pamukkale University, Denizli 20160, Turkey

**Keywords:** anti-Müllerian hormone, major depression, ovarian reserve, selective serotonin reuptake inhibitors

## Abstract

*Background and Objectives*: The goal of this study was to investigate the effect of selective serotonin reuptake inhibitor treatment on the ovarian reserves of women of reproductive age with major depressive disorder. *Materials and Methods*: The current study is a prospective controlled trial including 48 women with major depressive disorder and 48 age-matched healthy controls. Ovarian reserve tests are performed prior to treatment and after six cycles of selective serotonin reuptake inhibitor treatment in the major depressive disorder group. Serum follicle-stimulating hormone, luteinizing hormone, estradiol, and anti-Müllerian hormone levels were evaluated from blood samples, and endometrial thickness, total antral follicle count, and volume of both ovaries were assessed using transvaginal ultrasonography. *Results*: When the first measurements were compared, menstrual duration and menstrual bleeding increased (*p* = 0.007 and 0.005, respectively) and luteinizing hormone decreased (*p* = 0.045) in the major depressive disorder group, while follicle-stimulating hormone, estradiol, anti-Müllerian hormone, endometrial thickness, total antral follicle count, and mean ovarian volume did not differ significantly between groups (*p* > 0.05). When the major depressive disorder group’s first and final measurements were compared, follicle-stimulating hormone, estradiol, and endometrial thickness increased (*p* = 0.05, 0.0001, and 0.005, respectively), luteinizing hormone remained constant (*p* = 0.541), and anti-Müllerian hormone and total antral follicle count decreased (*p* = 0.024 and 0.042, respectively). *Conclusions*: In this study, we observed that the ovarian reserve test results of patients diagnosed with major depression for the first time after 6 months of SSRI treatment were significantly different from the results of the pretreatment and control groups.

## 1. Introduction

Depression affects 7.4% of adults in the United States between the ages of 18 and 39, with women (9.3%) being more affected than men (5.8%) [1]. Major depressive disorder (MDD) is a complicated condition that impacts multiple systems in the brain and peripheral nervous system, as well as the emotional and cognitive processes required for healthy daily functioning and quality of life. From puberty to old age, men’s new onset rates and 12-month prevalence of MDD remain relatively constant, whereas women’s rates and prevalence rise at puberty and remain higher than men’s until menopause [2]. According to research, depression is even more prevalent in infertile women [3]. Antidepressant medication is widely used to treat depression; antidepressants are currently being used by an estimated 9.2% of reproductive-age American women (18–39 years old) [4]. Selective serotonin reuptake inhibitors (SSRIs), such as citalopram, escitalopram, fluoxetine, paroxetine, fluvoxamine, and sertraline, are the most commonly used antidepressant medications [5]. 

The number and quality of oocytes present in a woman’s ovarian reserve (OR) determine her reproductive potential. Many tests have been studied to evaluate OR [6,7]. In women, it has been established that the highest level of anti-Müllerian hormone (AMH) expression was present in granulosa cells of secondary, preantral, and small antral follicles < or = 4 mm in diameter. In larger (4–8 mm) antral follicles, AMH expression gradually disappeared [8], and it is secreted prior to the follicle-stimulating hormone (FSH)-dependent selection of dominant follicles, so it is unaffected by FSH [8]. AMH may also shield developing follicles from premature maturation. Many studies show a link between serum AMH levels and the number of growing follicles. Furthermore, as women age, both AMH serum levels and the number of follicles decrease [9]. It is considered a valuable marker in the evaluation of OR due to its low variation between menstrual cycles [10]. Total antral follicle count (total AFC) is determined during the follicular phase of the cycle by transvaginal ultrasonography (USG) scanning of both ovaries from the outside to the inside and counting cystic structures sized between 2 and 10 mm without any anechoic solid images [10]. The two most commonly used OR markers are AMH and total AFC, both of which are substitutes for the actual OR [11]. During the follicular phase, the ovaries secrete estradiol (E2), and E2 levels vary between cycles but are typically less than 50 pg/mL on days 3–5 of the menstrual cycle. In the early follicular phase, high E2 levels (above 60–80 pg/mL) indicate reproductive aging and accelerated oocyte development [6]. Basal FSH is the simplest and most common test used to determine OR, measured between cycle days 2 and 4, and its value increases with age. An FSH value of 10–20 mIU/mL means a weak response to ovarian stimulation and a reduced chance of pregnancy [12].

Infertility rates have risen in recent years, paralleling the rise in depression rates among women of reproductive age. In addition, SSRIs are widely used in the treatment of depression. There are numerous reasons for the rise in infertility rates; we hypothesize that the presence of depression and the use of antidepressants is one of them. Therefore, in this study, we aimed to evaluate OR tests before and after antidepressant treatment in women of reproductive age who were diagnosed with depression for the first episode. The obtained data were compared with the data of the control group, consisting of healthy women. 

## 2. Materials and Methods

This case-controlled prospective study was carried out at Pamukkale University Hospital’s Obstetrics and Gynecology Clinic from November 2020 to July 2022, after ethics approval from the Pamukkale University Local Ethics Committee (13 October 2020; 19). Prior to taking part in the study, all participants gave written and informed consent.

### 2.1. Study Participants and Design

Women of reproductive age (18–35 years) who applied to the psychiatry clinic and were diagnosed with first-episode MDD using the Diagnostic and Statistical Manual of Mental Disorders, Fifth Edition (DSM 5) diagnostic criteria, and who were prescribed SSRI antidepressants, were referred to the study center of the gynecology clinic before treatment to be included in our study. In addition, healthy volunteer women of reproductive age (18–35 years) who applied to the gynecology outpatient clinic for a routine gynecological examination were referred to the study center to be included in the control group.

**Figure 1:**
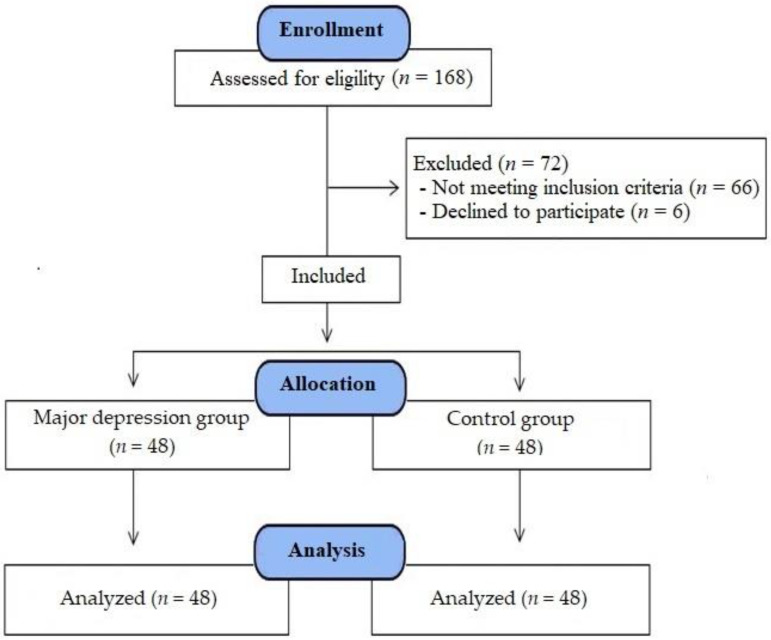
Flow consort chart of the study.

Exclusion criteria were: a previous history of SSRI use, a diagnosis of infertility, known serious chronic diseases (type 1 or 2 diabetes mellitus, Addison’s disease, Cushing syndrome, kidney failure, liver failure, thyroid dysfunction, etc.), urogenital tract infection, existing malignant diseases, polycystic ovarian syndrome (PCOS), use of anticoagulants, use of drugs that affect or alter the sex hormone profile, and alcohol use. A total of 168 women of reproductive age who were consecutively referred to the study center in the gynecology clinic were evaluated for study eligibility. Among them, three women with a diagnosis of infertility, nine women were diagnosed with pelvic pathologies (cervical or endometrial polyps, myomas, masses, or ovarian cysts), eight women with systemic diseases, seven women with acute infections, eight women who regularly used combination oral contraceptives (COCs), four women who used anticoagulants, 10 women whose treatment was changed due to drug side effects after starting SSRI antidepressant treatment, 12 women without regular menstrual cycles at the six-month follow-up after starting antidepressant therapy, five women who did not attend the follow-up examination after six cycles, and six women who later refused to participate were excluded. Thus, in total, 48 women with regular menstrual cycles (totaling 21–35 days) and who were diagnosed with MDD were assigned to the MDD group, while 48 women who had regular menstrual cycles (totaling 21–35 days) without depression or any known medical disease were assigned to the control group (Figure 1). 

Patients with regular medicament were included in the study. Sertraline, fluoxetine, and escitalopram were the SSRIs used in the patient group. The SSRI doses were used in accordance with the treatment guide and were also the most commonly used doses (50–100 mg/day for sertraline, 20–40 mg/day for fluoxetine, and 10–20 mg/day for escitalopram). In the MDD group, 40 cases were treated with sertraline, 5 with fluoxetine, and 3 with escitalopram. The Hamilton Depression Rating Scale (HAM-D) [13] was administered to the MDD group at baseline and 6 months. 

Participants were asked to complete a self-assessment questionnaire that recorded demographic information as well as clinical menstrual characteristics. Body mass index (BMI, in kg/m^2^) was calculated after height and weight were measured. 

### 2.2. Study Plan and Interventions

Patients diagnosed with MDD were called for control on the third to the fifth day of their cycles before beginning SSRI antidepressant treatment. After a 12-h fast, venous blood samples were collected on the third to the fifth day of their cycles. Serum FSH, luteinizing hormone (LH), E2, and AMH levels were measured in blood samples, and all transvaginal USGs were performed on the same day as the blood sampling. Endometrial thickness (ET), total AFC, and the volume of both ovaries were all measured using transvaginal USG. The ET was measured at the thickest point of the longitudinal segment. Follicle sizes were calculated by taking the average of the diameter measurements in three planes, and follicles with diameters ranging from 2 to 10 mm were considered antral. After measuring the diameters of each ovary in three vertical planes, the ovarian volumes were calculated using the formula D1 × D2 × D3 × 0.52. All cases in the MDD group started using SSRI antidepressants prescribed by the psychiatrist after the above examinations and evaluations were performed by the gynecologist. Following six cycles (roughly six months), on the third to the fifth day of their menstrual cycles, serum FSH, LH, E2, and AMH levels were re-evaluated after a 12-h fast, and the same researcher performed a transvaginal USG. All ET, total AFC, and ovarian volume measurements were repeated using transvaginal USG, and all results were recorded.

Following a 12-h fast, venous blood samples were collected from healthy volunteer women in the control group on the third to the fifth day of their cycles. The serum levels of FSH, LH, E2, and AMH were measured, and transvaginal USG was performed in all cases on the same day the serum samples were collected by the same researcher. As in the MDD group, following six cycles (roughly six months), on the third to the fifth day of their menstrual cycles, serum FSH, LH, E2, and AMH levels were re-evaluated after a 12-h fast, and the same investigator performed a transvaginal USG. Antidepressants were not prescribed because the control group consisted of healthy women who did not have MDD. 

### 2.3. Determination of Serum AMH Levels

Blood samples from the MDD and control groups were taken from the peripheral vein to determine serum AMH levels, which were measured using the Elecsys (Roche, Basel, Switzerland) AMH kit. The coefficients of variation for intra- and inter-assays were found to be 2.7 and 4.4%, respectively. 

### 2.4. Statistical Analysis

SPSS 25.0 was used to perform all statistical analyses. (IBM SPSS Statistics 25 software (Armonk, NY, USA: IBM Corp.)). Continuous variables were expressed using the mean, standard deviation (SD), and median (IQR: 25th–75th percentiles), while categorical variables were expressed using frequencies and percentages. Normality was determined using the Shapiro-Wilk test. When the parametric test assumptions were met, the independent samples test-test was used for group comparisons. When the parametric test assumptions were violated, the Mann—Whitney U test was used to compare independent groups. A parametric paired-samples test-test and a non-parametric Wilcoxon signed-rank test were used for pairwise comparisons; *p*-values less than 0.05 were considered statistically significant. The correlation coefficients and significance were calculated via the Spearman test in the evaluation of the relationships between the ordinal variables, at least one of which showed an abnormal distribution.

### 2.5. Ethical Approval

This study was carried out in accordance with the Helsinki Declaration’s principles. All participants provided written and informed consent prior to taking part in this study. The study was approved by the Pamukkale University Clinical Research Ethics Committee (13 October 2020; 19). 

## 3. Results

As shown in Table 1, there were no statistically significant differences between the groups in demographic characteristics such as age, BMI, parity (*p* = 0.355, 0.312, and 0.644, respectively), or menstrual length (*p* = 0.46). Before treatment, MDD patients had significantly higher menstrual duration (days) and menstrual bleeding (pads/day) than controls (*p* = 0.007 and 0.005, respectively).

In the FSH examinations, no statistically significant difference was found between the two groups in the first measurements (*p* = 0.063). In the final measurements, it was observed that the MDD group’s values were significantly higher than those of the control group (*p* = 0.006). In the in-group examinations, the MDD group showed a significant increase (*p* = 0.05), while the control group showed no statistically significant change (*p* = 0.679). Furthermore, no statistically significant difference in delta values (calculated by subtracting the final examination from the first examination; *p* = 0.265; Table 2) existed between the two groups. 

In the LH examinations, there was a statistically significant difference between the two groups in both the first and final measurements (*p* = 0.045 and 0.015, respectively). In both, it was observed that the values of the MDD group were significantly lower than those of the control group, but no significant change was observed in both groups following the in-group examinations (*p* = 0.722 and 0.541, respectively). Furthermore, no statistically significant difference in delta values existed between the two groups (*p* = 0.803; Table 2). 

In the E2 examinations, no statistically significant difference was found between the two groups in the first measurements (*p* = 0.125). In the final measurements, it was observed that the values of the MDD group were significantly higher than those of the control group (*p* = 0.021). In the in-group examinations, a significant increase was observed in the MDD group (*p* = 0.0001), while any change in the control group was not statistically significant (*p* = 0.278). In the delta values, the change in the MDD group was found to be significantly higher than in the control group (*p* = 0.0001; Table 2).

In the AMH examinations, no statistically significant difference was found between the two groups in the first measurements (*p* = 0.344). In the final measurements, it was observed that the values of the MDD group were significantly lower than those of the control group (*p* = 0.009). In the in-group examinations, a significant decrease was observed in the MDD group (*p* = 0.024), while any change in the control group was not statistically significant (*p* = 0.495). Furthermore, no statistically significant difference in delta values existed between the two groups (*p* = 0.172; Table 2). 

In the ET examinations, no statistically significant difference was found between the two groups in the first and final measurements (*p* = 0.884 and 0.125, respectively). In the in-group examinations, a significant increase was observed in the MDD group (*p* = 0.005), while any change in the control group was not statistically significant (*p* = 0.819). Furthermore, in terms of delta values, the change in the MDD group was significantly greater than in the control group (*p* = 0.047; Table 2).

In the total AFC examinations, no statistically significant difference was found between the two groups in the first measurements (*p* = 0.293). In the final measurements, it was observed that the values of the MDD group were significantly lower than those of the control group (*p* = 0.018). In the in-group examinations, a significant decrease was observed in the MDD group (*p* = 0.042), while any change in the control group was not statistically significant (*p* = 0.415). Furthermore, no statistically significant difference in delta values existed between the two groups (*p* = 0.552; Table 2). 

In the average ovarian volume examinations, no statistically significant difference was found between the two groups in the first measurements (*p* = 0.387). In the final measurements, it was observed that the values of the MDD group were significantly lower than those of the control group (*p* = 0.036). Furthermore, no significant difference was found between the two groups in the in-group examinations (*p* = 0.547 and 0.125, respectively), and the delta values did not differ statistically between the two groups (Table 2).

The relationship between delta values was obtained by subtracting the final examination from the first examination, and the clinical features were examined. While there was a statistically significant and positive relationship between the ET change and BMI values in the control group (*p* = 0.037), this relationship was not found in the MDD group (*p* = 0.597). While there was a statistically significant and positive relationship between the AMH change and BMI values in the control group (*p* = 0.023), this relationship was not observed in the MDD group (*p* = 0.176). In the control group, there was a statistically significant and positive relationship between the change in total AFC and BMI values (*p* = 0.038), and it was observed that the same relationship existed in the MDD group (*p* = 0.025). While there was a statistically significant and positive relationship between ovarian volume change and BMI values in the control group (*p* = 0.031), this relationship was not observed in the MDD group (*p* = 0.261), and while there was a statistically significant and positive relationship between FSH change and menstrual length in the MDD group (*p* = 0.019), this relationship was not found in the control group (*p* = 0.286; Table 3).

According to the psychiatrist who examined the MDD group, the patients in the MDD group had a mean HAM-D score of 23.8 before treatment and 7.9 after six months of treatment. This difference in HAM-D scores before and after treatment was statistically significant (*p* < 0.001; Table 4). In addition, the treatment success of our MMD group patients who were diagnosed with major depression for the first time after 6 months of SSRI treatment was too good to be true.

## 4. Discussion

In the current study, we aimed to evaluate whether SSRI group antidepressants, which are widely used in the treatment of depression, adversely affect OR in women of reproductive age. Our main findings were that LH levels were significantly lower in the MDD group, menstrual duration and bleeding were significantly higher in the control group, FSH and E2 levels were significantly higher, and AMH and total AFC measurements were significantly lower in the MDD group after SSRI treatment compared to pretreatment.

Inhibin B and AMH are glycoprotein hormones produced by small ovarian follicles. Inhibin B is primarily secreted by preantral follicles, whereas AMH is secreted by primary, preantral, and early antral follicles. As the number of ovarian follicles decreases with age, so do AMH and early-follicular-phase inhibin B concentrations. Reduced inhibin B secretion reduces the level of central negative feedback, which leads to increased pituitary FSH secretion and higher late-luteal and early-follicular FSH concentrations (an indirect measure). In turn, the earlier increase in FSH levels stimulates an earlier onset of new follicular growth and an increase in E2 concentrations, ultimately shortening the follicular phase and the overall cycle. These hormone tests are, therefore, used as markers of ovarian reserve [10,14]. 

Multiple studies [15,16] have found that reproductive hormones have an impact on depression. Depression, on the other hand, influences the regulation of reproductive hormones. The hypothalamic-pituitary-adrenal (HPA) axis becomes activated during stress, causing the hypothalamus to secrete corticotropin-releasing hormone (CRH). CRH causes the pituitary gland to release adrenocorticotropic hormone (ACTH), which then interacts with the adrenal cortex and causes cortisol to be released. In MDD, chronic stress disrupts the HPA axis [17], and GnRH neurons, gonadotrophs, and the gonads are all inhibited by stress-induced glucocorticoids [18]. Furthermore, elevated CRH levels in depression inhibit the HPG axis [19,20]. According to previous research, up to 41% of women seeking fertility treatment [21] and 49.1% of male in vitro fertilization (IVF) patients [22] suffer from depression. In the original HSMC study [23], 332 women with a history of depression at baseline experienced an earlier onset of perimenopause, with perimenopause described as changes in menstrual cycle regularity and bleeding patterns. Additionally, these women had higher serum FSH and LH levels, as well as lower E2 levels, than those who were not depressed. In our current study, when the pretreatment evaluations of the MDD group cases were compared with the first evaluations of the control group cases, there was no significant difference among FSH, E2, and AMH levels, while LH levels in the MDD group were significantly lower. Likewise, when the first ultrasonographic images of the MDD group and control group cases were compared, no significant difference was found between the two groups in the ET, total AFC, and average ovarian volume measurements. We think the reason for the isolated LH decrease in the MDD group cases may be due to the early diagnosis of the patients in this group, who were diagnosed with MDD for the first time, as well as the short exposure time to MDD. This result supports the thesis that LH is the first hormone to be affected in the presence of MDD. In addition, when we investigated menstrual characteristics in the first case evaluations in our study, while menstrual duration and menstrual bleeding were found to be significantly higher in the MDD group, there was no significant difference between the two groups in terms of menstrual length. We believe the reason for this increase in menstrual duration and menstrual bleeding in the MDD group may be due to the effect of increased ACTH in depression and, therefore, the effect of cortisone, while the stress situation in depression may have increased due to an increase in blood bradykinin and prostaglandin levels, causing enlargement of the pelvic blood vessels and an increase in blood flow [14].

Because of their consistent efficacy and favorable safety profile, SSRIs are now widely used in the treatment of a variety of psychiatric disorders. These drugs, however, can have serious side effects such as gastrointestinal bleeding and sexual dysfunction [24,25]. In this context, in men, SSRIs have been linked to decreased libido, erectile dysfunction, and delayed ejaculation, while in women they have been linked to genital anesthesia, lubrication loss, and anorgasmia [25]. For a long time, the sexually adverse effects of SSRIs were thought to be reversible because the symptoms went away quickly after the medication was stopped. However, there has been an increase in reports of sexual dysfunction that lasts for several years after SSRI discontinuation in recent years [26]. Few studies have been conducted to examine the impact of antidepressants on female fertility or fecundity [27,28]. According to one cohort study of women early in their fertility attempts (<3 months), antidepressant use was associated with lower fecundability in any given cycle, regardless of the patient’s depression history [27]. In contrast, a cohort study of women attempting to conceive discovered an inverse relationship between depressive symptoms and fertility, even though antidepressants did not affect the likelihood of conceiving [28]. Predictably, most studies investigating the effect of SSRIs on fertility were conducted in infertile patients [29,30,31,32]. One randomized controlled trial found no differences in anxiety measures or IVF outcomes between groups when patients were given fluoxetine or folic acid (placebo) during the IVF process [29]. However, the patients were given fluoxetine for an average of 26 days, which may be insufficient time to detect an effect. A large cohort study of over 23,000 nulliparous women using Swedish National Register data examined the relationship between anxiety and depression, antidepressant use, and IVF outcomes [30], and the researchers discovered no statistically significant link between SSRI use and IVF outcomes. Furthermore, a large, retrospective cohort study of IVF patients examined clinical outcomes as well as aneuploidy status in couples who had their embryos genetically tested prior to embryo transfer [31]. In over 2000 cycles assessed, there were no differences in IVF outcomes between SSRI users and non-users, including the rates of implantation and clinical pregnancy. AMH levels, a marker of OR, were lower in the SSRI-exposed group regardless of age, implying that women exposed to SSRIs had a lower baseline OR. In the SSRI group, there were also trends toward lower peak estrogen levels and higher rates of early pregnancy loss, but these results were not statistically significant. Finally, in a large cohort study with two randomized, controlled trials for the treatment of infertility in PCOS and unexplained infertility, the effects of non-IVF fertility treatments on maternal MDD, antidepressant use, and paternal MDD were investigated [32]. The study found that maternal MDD was unrelated to pregnancy outcomes, which included not only live birth rates but also conceptions and first-trimester pregnancy losses. Although differences in live birth rates were not associated with antidepressant use, maternal antidepressant use was associated with a higher rate of early miscarriage. In our current study, when the hormonal parameters of the MDD group patients before antidepressant treatment and in the sixth cycle of treatment were compared, FSH and E2 values increased, AMH values decreased, and LH values did not change after treatment. Likewise, when we compared ultrasonographic parameters, ET measurements increased, total AFC measurements decreased, and average ovarian volume measurements did not change after treatment. These results of our study show that SSRI treatment negatively affects OR in women of reproductive age with a diagnosis of MDD. The decrease in total AFC and AMH, as well as the increase in FSH, suggest that these adverse effects of SSRIs on OR may be due to their direct effects on ovarian tissue rather than GnRH inhibition. In addition, according to the results of our study, it was observed that ET measurements increased after treatment in MDD cases, and we think this increase may be due to the increase in E2 levels. We hypothesize that the reason for this increase in E2 levels may be due to increased FSH levels, increasing E2 production in large follicles. In addition, one of the reasons for the decrease in AMH levels may be these increased E2 levels. Recent findings that E2 inhibits AMH expression via the estrogen receptor ß could reconcile this result [33]. FSH stimulates AMH production first and then in more mature follicles, and stimulates E2 secretion, which inhibits AMH synthesis.

Some limitations of the present study should be considered. The following study limitations were identified: (1) this was a single-center study; (2) a group with a diagnosis of infertility was not included; (3) a group of patients with untreated major depression was not included; (4) a group with irregular menstrual cycles after SSRI treatment was not included; (5) cases with leiomyoma, endometrioma, and PCOS were not evaluated; and (6) cases were assessed after only six months. 

## 5. Conclusions

In conclusion, the rise in infertility rates paralleled by the rise in depression rates among women of reproductive age has led us to believe that there may be a link between these two conditions. In the current study, pretreatment LH levels and menstrual characteristics of non-infertile depressive women were significantly altered compared to the control group. In addition, the FSH, E2, and AMH levels, as well as ET and total AFC measurements of depressive women after SSRI treatment, were significantly altered compared to pretreatment. Because we were unable to establish a patient group with untreated major depression, we cannot determine whether these changes were caused by major depression or by SSRIs. However, our findings suggest that SSRIs may also affect these outcomes. More research is needed to confirm our findings. 

## Figures and Tables

**Table 1:** Differences in demographic characteristics according to the study group.

	Control Group		MDD Group		
	Mean ± S.D	Med (IQR)	Mean ± S.D	Med (IQR)	Inter Group *p*
Age	27.69 ± 3.38	28 (24.25–30)	28.29 ± 3.65	29 (24.25–31.75)	0.355 (z = −0.926)
BMI (kg/m^2^)	27.54 ± 3.28	27.3 (24.35–30.2)	28.29 ± 3.32	28.25 (25.38–31.28)	0.312 (z = −1.012)
Parity	1.08 ± 0.96	1 (0–2)	0.98 ± 0.89	1 (0–2)	0.644 (z = −0.463)
Menstrual cycle length (days)	27.54 ± 1.86	28 (26–29)	27.81 ± 1.76	28 (26–29)	0.46 (z = −0.739)
Menstrual cycle duration (days)	4.81 ± 1.82	5 (3–6)	5.83 ± 1.74	6 (4–7)	0.007 * (z = −2.699)
Menstrual bleeding (pads/day)	5.27 ± 1.72	5 (4–7)	6.29 ± 1.91	6 (5–8)	0.005 * (z = −2.786)

* *p* < 0.05 statistically significant; S.D—Standard Deviation; Med (IQR)—Median (25th–75th percentiles); z—Mann—Whitney U test; MDD group—Major depressive disorder group; BMI—Body mass index.

**Table 2:** Differences and changes in measurements according to the study group.

	Control Group		MDD Group		
	Mean ± S.D	Med (IQR)	Mean ± S.D	Med (IQR)	Inter Group *p*
First ET (mm)	6.25 ± 1.72	6 (5–8)	6.17 ± 1.55	6 (5–8)	0.884 (z = −0.146)
Final ET (mm)	6.21 ± 1.71	6 (5–7)	6.81 ± 1.9	7 (5–9)	0.126 (z = −1.53)
Delta ET (mm)	0.04 ± 1.43	0 (−1–1)	−0.65 ± 1.45	−1 (−1–0.75)	0.047 * (z = −1.99)
Intra Group *p*	0.819 (z = −0.228)		0.005 * (z = −2.799)		
First E2 (ng/L)	56.54 ± 13.91	56 (46.25–69)	51.83 ± 12.83	53.5 (39.5–61.75)	0.125 (z = −1.536)
Final E2 (ng/L)	54.44 ± 15.62	56.5 (40.25–62.75)	60.13 ± 11.81	63 (53–69)	0.021 * (z = −2.314)
Delta E2 (ng/L)	2.1 ± 13.29	2 (−7.75–10)	−8.29 ± 7	−9 (−14–−4)	0.0001 * (t = 4.796)
Intra Group *p*	0.278 (t = 1.097)		0.0001 * (t = −8.212)		
First FSH (mIU/mL)	7.21 ± 1.46	7.5 (6–8)	7.79 ± 1.37	8 (7–9)	0.063 (z = −1.862)
Final FSH (mIU/mL)	7.33 ± 1.6	8 (6–9)	8.25 ± 1.33	8.5 (7–9)	0.006 * (z = −2.768)
Delta FSH (mIU/mL)	−0.13 ± 2.08	0 (−2–1)	−0.46 ± 1.56	−1 (−1–1)	0.265 (z = −1.115)
Intra Group *p*	0.679 (t = −0.416)		0.05 * (z = −1.954)		
First LH (mIU/mL)	6.02 ± 1.47	6 (5–7)	5.38 ± 1.52	5 (4–6)	0.045 * (z = −2.001)
Final LH (mIU/mL)	5.88 ± 1.28	6 (5–7)	5.25 ± 1.06	5 (4–6)	0.015 * (z = −2.432)
Delta LH (mIU/mL)	0.15 ± 1.62	0 (−1–1)	0.13 ± 1.28	0.5 (−1–1)	0.803 (z = −0.249)
Intra Group *p*	0.722 (z = −0.356)		0.541 (z = −0.611)		
First AMH (ng/mL)	3.95 ± 1.45	4.2 (2.4–5.18)	3.69 ± 1.53	3.7 (2.15–4.88)	0.344 (z = −0.946)
Final AMH (ng/mL)	3.82 ± 1.27	3.6 (2.95–4.88)	3.15 ± 1.21	3.1 (2.13–3.95)	0.009 * (z = −2.623)
Delta AMH (ng/mL)	0.13 ± 1.3	0.15 (−0.68–1.1)	0.54 ± 1.6	0.35 (−0.5–1.45)	0.172 (t = −1.378)
Intra Group *p*	0.495 (t = 0.688)		0.024 * (t = 2.334)		
First TotalAFC	9.56 ± 1.67	10 (8–11)	9.21 ± 1.64	9 (8–10)	0.293 (z = −1.052)
Final TotalAFC	9.38 ± 1.52	9.5 (8–10)	8.65 ± 1.3	9 (8–9.75)	0.018 * (z = −2.374)
Delta AFC	0.19 ± 1.58	0 (−1–1)	0.56 ± 1.82	0 (−1–2)	0.552 (z = −0.594)
Intra Group *p*	0.415 (t = 0.822)		0.042 * (z = −2.034)		
First Average ovarian volume (mm^3^)	6.23 ± 1.12	6.1 (5.3–7.05)	6.06 ± 1.09	6 (5.1–6.7)	0.387 (z = −0.865)
Final Average ovarian volume (mm^3^)	6.14 ± 1.02	6.1 (5.3–6.78)	5.66 ± 0.82	5.4 (5.13–6.28)	0.036 * (z = −2.092)
Delta Average ovarian volume (mm^3^)	0.09 ± 1.07	0 (−0.68–0.88)	0.39 ± 1.23	0.05 (−0.48–1.23)	0.422 (z = −0.803)
Intra Group *p*	0.547 (t = 0.607)		0.125 (z = −1.535)		

* *p* < 0.05 statistically significant; S.D—Standard Deviation; Med (IQR)—Median (25th–75th percentiles). For inter-group examinations: t—Independent Samples *t*-test; z—Mann—Whitney U test. For intra-group examinations: t—Paired samples *t*-test; z—Wilcoxon Signed Rank Test; Delta—Pre-post test change values (difference); MDD group—Major depressive disorder group; ET—Endometrial thickness; E2—Estradiol; FSH—Follicle-Stimulating Hormone; LH—Luteinizing Hormone; AMH—Anti-Müllerian Hormone; AFC—Antral Follicle Count.

**Table 3:** Relationships among examined parameters.

			ET Delta	E2 Delta	FSH Delta	LH Delta	AMH Delta	AFC Delta	AOV Delta
Control group	Age (year)	r	−0.09	−0.279	0.167	0.053	−0.195	−0.173	−0.184
		*p*	0.541	0.055	0.258	0.718	0.184	0.24	0.21
	BMI (kg/m^2^)	r	0.302 *	−0.184	−0.248	−0.132	0.327 *	0.300 *	0.312 *
		*p*	0.037	0.211	0.09	0.373	0.023	0.038	0.031
	Parity	r	−0.077	−0.265	−0.043	−0.06	0.006	0.011	−0.02
		*p*	0.604	0.069	0.77	0.686	0.967	0.94	0.89
	Menstrual cycle length (days)	r	0.012	0	−0.157	0.07	0.206	0.102	0.158
		*p*	0.938	0.999	0.286	0.635	0.161	0.49	0.285
	Menstrual cycle duration (days)	r	−0.039	−0.134	−0.076	−0.15	0.046	0.177	−0.041
		*p*	0.794	0.362	0.608	0.309	0.757	0.23	0.784
	Menstrual bleeding (pads/day)	r	0.106	−0.217	−0.139	−0.13	0.121	0.133	0.034
		*p*	0.473	0.138	0.346	0.379	0.411	0.369	0.819
MDD group	Age (year)	r	−0.033	−0.168	−0.047	0.054	0.037	0.034	0.023
		*p*	0.826	0.255	0.751	0.714	0.801	0.817	0.877
	BMI (kg/m^2^)	r	0.078	−0.031	−0.165	−0.013	0.199	0.324 *	0.166
		*p*	0.597	0.833	0.262	0.929	0.176	0.025	0.261
	Parity	r	0.058	−0.201	−0.021	0.161	0.042	−0.004	0.032
		*p*	0.693	0.171	0.887	0.273	0.776	0.979	0.827
	Menstrual cycle length (days)	r	0.053	−0.009	0.338 *	0.196	−0.255	−0.224	−0.195
		*p*	0.72	0.951	0.019	0.183	0.08	0.125	0.184
	Menstrual cycle duration (days)	r	−0.259	0.092	0.039	0.027	0.268	0.229	0.188
		*p*	0.075	0.535	0.791	0.855	0.066	0.118	0.202
	Menstrual bleeding (pads/day)	r	−0.165	0.112	0.099	0.101	0.165	0.178	0.145
		*p*	0.262	0.449	0.504	0.496	0.262	0.225	0.325

* *p* < 0.05 statistically significant correlation; r—Spearman correlation coefficient; MDD group—Major depressive disorder group; ET—Endometrial thickness; E2—Estradiol; FSH—Follicle-Stimulating Hormone; LH—Luteinizing Hormone; AMH—Anti-Müllerian Hormone; AFC—Antral Follicle Count; AOV—Average ovarian volume; BMI—Body mass index.

**Table 4:** HAM-D scores before and after treatment in the MDD group.

	HAM-D Scores (before Treatment)		HAM-D Scores (after Treatment)		
	Mean ± S.D	Med (IQR)	Mean ± S.D	Med (IQR)	*p*
MDD Group (*n* = 48)	28.3 ± 4.1	28 (24–32)	8.4 ± 2.8	8 (5–12)	<0.001 *

* *p* < 0.05 statistically significant; S.D—Standard Deviation; Med (IQR)—Median (25th–75th percentiles); HAM-D—Hamilton Depression Rating Scale; MDD group—Major depressive disorder group.

## Data Availability

The datasets used and/or analyzed during the current study are available from the corresponding author upon reasonable request.
